# Tissue probability-based AC for neurological PET/MR using SPM8

**DOI:** 10.1186/2197-7364-2-S1-A26

**Published:** 2015-05-18

**Authors:** Jarmo Teuho, Jani Linden, Jarkko Johansson, Jouni Tuisku, Terhi Tuokkola, Mika Teras

**Affiliations:** Turku PET Centre, Turku, Finland

The quantitative accuracy of brain PET/MR has been reported to be reduced compared to PET/CT due to difficulties related to bone in MR-based attenuation correction (MRAC), especially in regions near the skull. To reduce these effects, we designed an offline, tissue probability-based (TPB-AC) using SPM8, which includes soft tissue, air and bone. Thus, both the increased attenuation due to skull and the reduced attenuation due to sinuses are taken into account. The method was developed to use the data collected with the standard anatomical MRAC consisting of 3D T1 FFE, with isotropic voxel size of 2 mm. Thus, collection of additional sequences or modifications to the standard attenuation correction sequence are not required. Data from F18-FDG brain studies performed both with PET/CT and PET/MR was used in AC validation. Seven patients were reconstructed with the standard attenuation map, TPB-AC and CTAC in PET/MR. Regional analysis between attenuation corrected PET images was conducted using CTAC as reference. Using TPB-AC, the relative difference in seven subjects when compared to CTAC in all regions was -3 %, -2 %, -5 %, 4 %, -3 %, 1 %, 0 %. In clinical AC the difference was -9 %, -8 %, -9 %, -3 %, -10 %, -5 %, -6 %. Region-wise, the benefit from TPB-AC was most pronounced in precentral, superior frontal and middle frontal gyrus where underestimation from clinical AC of: -11 %, -12 % and -12 % was reduced to: -1%, -2 % and -1 % when using TPB-AC. Tissue probability-based AC was deemed a promising interim method for PET/MR AC until standardized, commercialized solutions become widely available. To test the robustness of the method, patients with dental implants or deformed anatomy would have to be included in the study group.

